# Electrochemically derived functionalized graphene for bulk production of hydrogen peroxide

**DOI:** 10.3762/bjnano.11.34

**Published:** 2020-03-09

**Authors:** Munaiah Yeddala, Pallavi Thakur, Anugraha A, Tharangattu N Narayanan

**Affiliations:** 1 Tata Institute of Fundamental Research - Hyderabad, Sy. No. 36/P, Gopanapally Village, Serilingampally Mandal, Hyderabad 500107, India

**Keywords:** electrochemical oxygen reduction, functionalized carbon, functionalized graphene, H_2_O_2_ production, water treatment

## Abstract

On-site peroxide generation via electrochemical reduction is gaining tremendous attention due to its importance in many fields, including water treatment technologies. Oxidized graphitic carbon-based materials have been recently proposed as an alternative to metal-based catalysts in the electrochemical oxygen reduction reaction (ORR), and in this work we unravel the role of C=O groups in graphene towards sustainable peroxide formation. We demonstrate a versatile single-step electrochemical exfoliation of graphite to graphene with a controllable degree of oxygen functionalities and thickness, leading to the formation of large quantities of functionalized graphene with tunable rate parameters, such as the rate constant and exchange current density. Higher oxygen-containing exfoliated graphene is known to undergo a two-electron reduction path in ORR having an efficiency of about 80 ± 2% even at high overpotential. Bulk production of H_2_O_2_ via electrolysis was also demonstrated at low potential (0.358 mV vs RHE), yielding ≈34 mg/L peroxide with highly functionalized (≈23 atom %) graphene and ≈16 g/L with low functionalized (≈13 atom %) graphene, which is on par with the peroxide production using state-of-the-art precious-metal-based catalysts. Hence this method opens a new scheme for the single-step large-scale production of functionalized carbon-based catalysts (yield ≈45% by weight) that have varying functionalities and can deliver peroxide via the electrochemical ORR process.

## Introduction

Hydrogen peroxide (H_2_O_2_) is identified as one among the most important 100 chemicals in the world, and its applications extend from the pharmaceutical industry to water purification [1–3]. Today, a majority of the required H_2_O_2_ is produced through the complex and energy-intensive anthraquinone method [4], and although it is popular, it has drawbacks such as side reactions, which consume the catalyst leading to the regeneration and hydrogenation of the catalyst [4]. Alternative routes for peroxide generation include direct preparation of H_2_O_2_ from oxygen and hydrogen, oxidation of alcohols [5], photocatalysis [6], and electrochemical processes such as the electro-Fenton process [7], microbial electrosynthesis [8], and proton exchange membrane (PEM) assisted synthesis [9]. Further, in situ generation of peroxide from dissolved oxygen via electrochemical ORR is identified as an effective route for water treatment technology, where economically viable, biologically friendly, sustainable catalysts are required [10–14].

Of the various routes, the direct synthesis of H_2_O_2_ through the reaction between H_2_ and O_2_ in the presence of a catalyst [15] is one of the possible routes having high yield, while the direct mixing of H_2_ and O_2_ can be explosive in nature [16], and hence requires a large quantity of another gas such as N_2_ and CO_2_ to dilute the reactant gases [17]. Electrochemical synthesis methods such as the PEM fuel cell reactor-based method can overcome this limitation; however, this method relies on expensive membranes to separate hydrogen and oxygen and to directly yield H_2_O_2_ from them [18]. Later, this method was modified by generating protons (H^+^) through water oxidation which eliminated the direct purging of H_2_ gas [9]. The major roadblock in this method is the development of a sustainable electrocatalyst for the selective reduction of oxygen to H_2_O_2_ [19–23]. Today, most electrochemical H_2_O_2_ production methods rely on precious-metal-based materials or transition metal and/or metal oxides, and hence their economic viability for the future technologies is highly questionable [10,24–27]. Hence new metal-free electrode materials for H_2_O_2_ generation are highly sought after for future technologies.

Recently, carbon-based catalysts have emerged as an alternate material for existing metal-based technologies [28–29]. For example, carbon nanotubes (CNTs) have been well studied for their catalytic activity, although conflicting reports exist due to the presence of unavoidable metallic impurities present [30–33]. With the emergence of graphene, heteroatom doping in sp^2^ graphitic structures is found to be an engineering pathway for altering the inert catalytic activity of planar honeycomb lattices of graphene and its derivatives [34–35]. It has been found that certain heteroatoms doped into graphene can even outperform the benchmark catalysts such as platinum (Pt/C) in their long-run alkaline ORR process where the extended stability in electrochemical processes is one of the crucial issues with Pt/C [36]. In all of these doped systems, carbon atoms near to the defect centers are found as catalytically active centers [37]. Recently, an ultra-small amount of selenium (Se) edge functionalized graphene (reduced graphene oxide (rGO)) was found to undergo a direct four-electron path ORR process in alkaline medium, where rGO undergoes a two-electron path peroxide route ORR [35]. In this process, Se acts as a single atom site catalyst. In a nutshell, depending on the nature of the dopant and its position in the host lattice, it has been well reported that one can engineer the electrochemical activity of nanographitic systems and the catalytic reaction pathways [31,38–40].

Very recently, oxidized graphitic structures were identified for their efficacy towards the alkaline ORR process leading to selective peroxide production. In a recently reported theoretical study by Cui et al., the carbon atoms near the oxygen functionalities are used for their ORR efficacy via a two-electron pathway [37]. It was further experimentally shown that carbon materials such as CNTs, graphene, etc. can be oxidized via chemical treatment, and these oxidized forms of sp^2^–sp^3^ carbon systems prefer peroxide formation in alkaline ORR process [25]. Such studies are supported by reports from other groups, where McCloskey et al. showed that sp^2^-hybridized carbon near-ring ether defects along sheet edges are the most active sites for peroxide production in rGO [3]. They also showed that the performance of these rGO-based catalysts in alkaline conditions under low overpotential outperform the existing state-of-the-art catalysts. However, a large extent of oxidation may hamper the charge transfer properties of functionalized graphene (graphene oxide (GO) or other functional derivatives of graphene) [41]. Hence the single-step method for the production of large scale, controllably functionalized graphene is of high demand, and in this work, we demonstrate such a method to control the extent of oxidation. Further, although it was proposed that C=O (quinone) functional groups are the major candidates in deciding the peroxide route O_2_ reduction, here we provide experimental evidence for tuning the quinone groups in graphene and its correlation to the peroxide production.

In one of our previous works, different halogenated graphene materials were developed via a single-step electrochemical exfoliation (EE) method [42]. It was found that such a method can produce graphene with varying degrees of oxygen functionalities [43]. Here we explore the possibility of functional groups to control graphene toward the electrochemical production of H_2_O_2_ in alkaline medium, and the amount of peroxide generated is quantified using a spectroscopic technique. A large amount of H_2_O_2_ is found to be formed via such simple modification of the exfoliation parameters, and the details are discussed in this article.

## Results and Discussion

**Figure 1:**
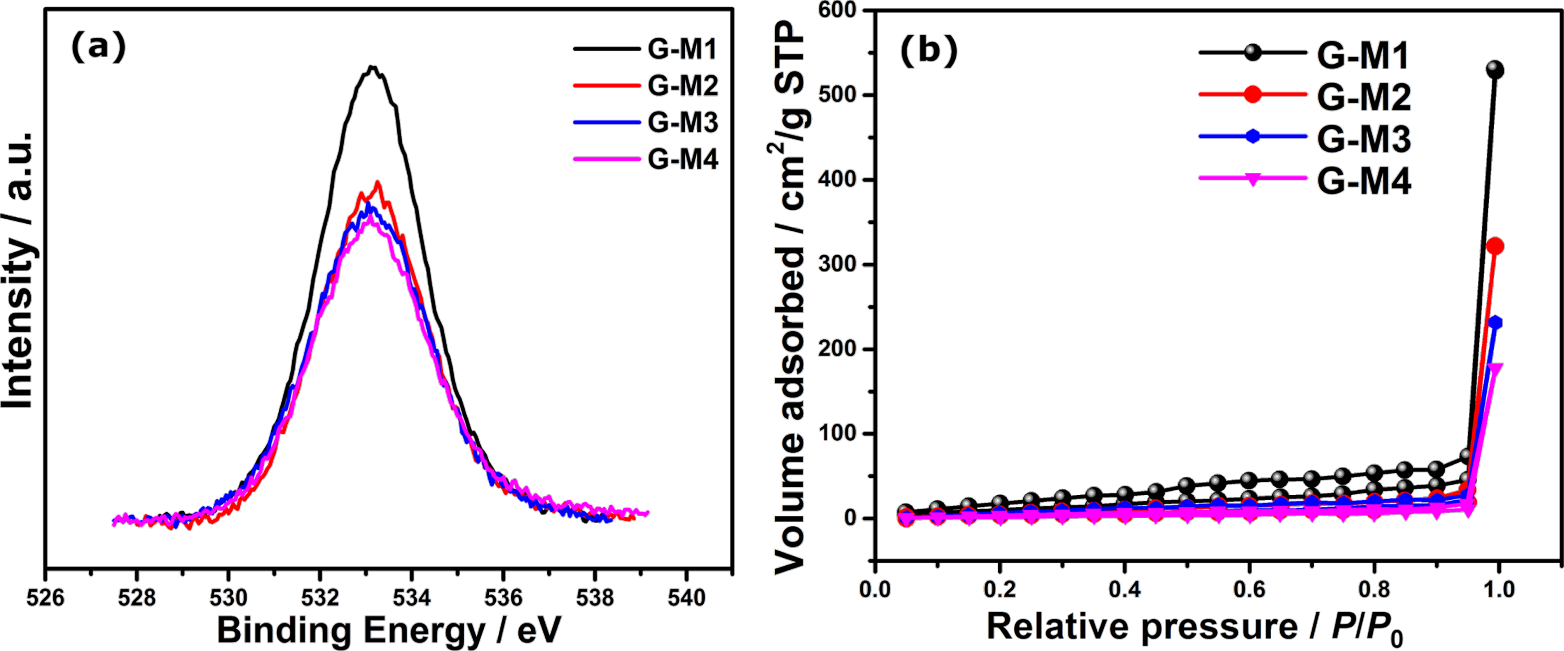
(a) High resolution O 1s XPS spectra of different EEG samples. (b) BET isotherms of different EEG samples.

The detailed physical characterization of electrochemically exfoliated graphene (EEG) was given in our recent report, where the variation in the oxygen functionalities, amount of fluorine content in exfoliated graphene, etc. were shown [43]. As discussed previously, the surface oxygen functional groups are crucial for the reduction of molecular oxygen to H_2_O_2_ and hence high-resolution O 1s X-ray photoelectron spectroscopy (XPS) was carried out. The O 1s peaks of different EEG samples are shown in Figure 1a. It can be seen that the intensity of the O 1s peak decreases from G-M1 to G-M4 (where G refers to graphene and M1, M2, M3, and M4 refer to the respective molarity of the electrolyte used for the exfoliation), and the elemental composition calculated from the survey spectrum of the materials revealed that the degree of oxygen functionalization varies from ≈21 to ≈10 atom % from G-M1 to G-M4 (as inferred from the XPS survey spectrum as well as the high-resolution C 1s and O 1s XPS spectra (Supporting Information File 1, Figure S1a)) [43]. The O 1s spectrum can be deconvoluted into two distinct peaks (as shown in Figure S2) centered at 532.2 eV and 533.4 eV, corresponding to alcoholic (C–OH)/ether type of oxygen in ester functional groups and carbonyl (C=O in –COOH) functional groups, respectively [42,44]. Interestingly, irrespective of the degree of functionalization (oxygen content), carbonyl groups are found to be the major component in all the EEG samples. This correlates with the Fourier-transform infrared spectroscopy (FTIR) based analysis (Figure S1b), which also shows the presence of covalent C–F functional groups in all the samples. The samples also contain fluorine as one of the dopants with a content varying from 2.3 to 3.9 atom %. However, previous studies showed that the fluorine-doped graphene systems follow a direct four-electron ORR path [45–48], whereas some of the other fluorine-doped carbon prefers the H_2_O_2_ path during the ORR [49–50]. Along with the degree of functionalization, the surface area of the material can also influence the catalytic property of the materials since these EEG samples are derived from bulk graphite using a single-step exfoliation in different electrolytes. The BET isotherms of the EEG samples are shown in Figure 1b. The shape of the nitrogen adsorption and desorption curves displays a typical type III behavior, which corresponds to that of a layered material [42,51]. The surface area varies from 46 ± 2 m^2^/g (G-M1) to 11 ± 2 m^2^/g (G-M4). The change in the surface area can be attributed to the rate of exfoliation of the graphite rod which in turn depends upon the availability of fluoride and hydroxide ions at the anode (i.e., the higher the hydroxide ions the faster the exfoliation), which is in line with our recent report [43].

As described earlier, transmission electron microscopy (TEM) images of EEG samples indicate that the average lateral size is about 3–5 µm (more images in Supporting Information File 1) and scanning electron microscopy (SEM) images indicate the formation of randomly oriented exfoliated graphene nanosheets [43]. However, the TEM images show that the thickness of the graphene increases from G-M1 to G-M4, where the G-M4 is found to block the electron beam despite its layered nature (Supporting Information File 1, Figure S2). Here the thickness variation is confirmed using atomic force microscopy (AFM), and the results are given in Figure S3. This indicates that with an increase in the concentration of the electrolyte, the thickness is increased from 40 nm to 140 nm (Figure S3), which corresponds with the TEM analysis [43] and BET-based surface area data. Hence from the TEM, BET, and AFM analysis, it can be concluded that the electrolyte concentration is important for the electrochemical exfoliation assisted synthesis of ultrathin graphene layers, and the electrolyte concentration also determines the extent of functionalization of graphene.

**Figure 2:**
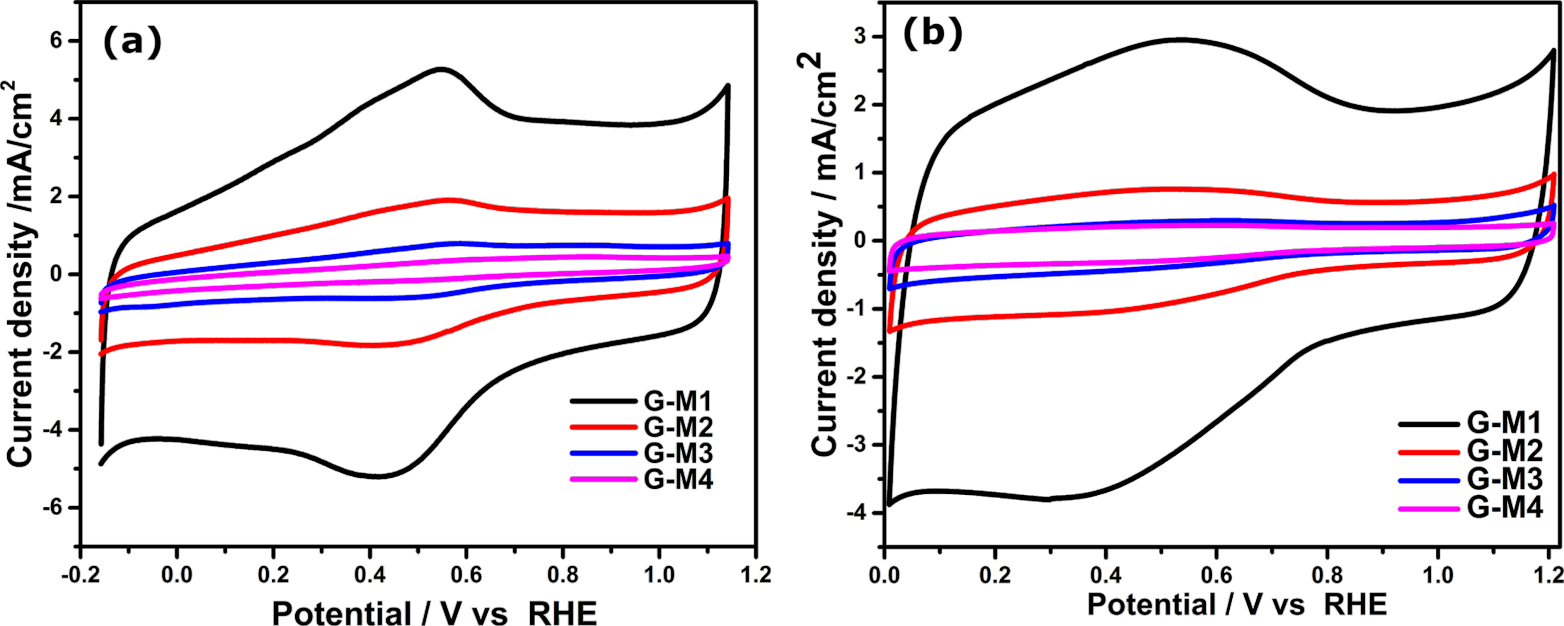
The CV profiles of different EEG samples in (a) acidic (0.5 M H_2_SO_4_) and (b) alkaline (0.1 M KOH) electrolyte at 100 mV/s scan rate. The CV with low scan rate (5 mV/s) is shown in Supporting Information File 1, Figure S4a. The current densities are calculated using the geometrical area.

The presence of the C=O groups is further confirmed by cyclic voltammetry (CV) measurements in alkaline and acidic electrolyte. The CV profiles of the EEG samples (EEG-modified glassy carbon electrode (GCE) as the working electrode in a three-electrode set up) in the acidic and alkaline medium between −0.2 to 1.2 V vs RHE at 100 mV/s scan rate are shown in Figure 2. Two distinct features observed in the CV profiles are: the difference in the area under the curve of the different EEG samples, which indicates the difference in the surface area of the electrodes; and secondly, the Faradaic redox peaks in acidic CV curves, which is nearly absent under alkaline conditions. Under both conditions (alkaline and acidic), G-M1 shows the highest surface area and G-M4 showed the lowest. This systematic variation in the electrochemical surface area corresponds with the BET analysis.

As mentioned, the CV profiles in the acidic medium show redox peaks at ≈0.5 V (0.5 M H_2_SO_4_), which corresponds to the redox reaction of oxygen functional groups such as quinone to hydroquinone, as per the following equation [52–54]:

[1] $$\text{Q}+2{\text{H}}^{+}↔{\text{QH}}_{2}$$

Interestingly, the intensity of the peak increases with the degree of functionalization, which further supports the assumption that the redox peak at 0.5 V is due to functionalization. These results are in good agreement with XPS data, as discussed previously. These redox peaks are not observed (only broad peaks) in alkaline medium (Figure 2b) under identical conditions due to the lack of the supply of protons (in acidic media, the conversion is 2H^+^, 2e^−^ reduction while in alkaline media it is only 2e^−^ reduction where Q^2−^ stabilized by water molecules and all species Q^2−^, QH^−^, QH^2−^ are present in equilibrium). This indicates the presence of C=O groups and their role in electrochemical processes.

**Figure 3:**
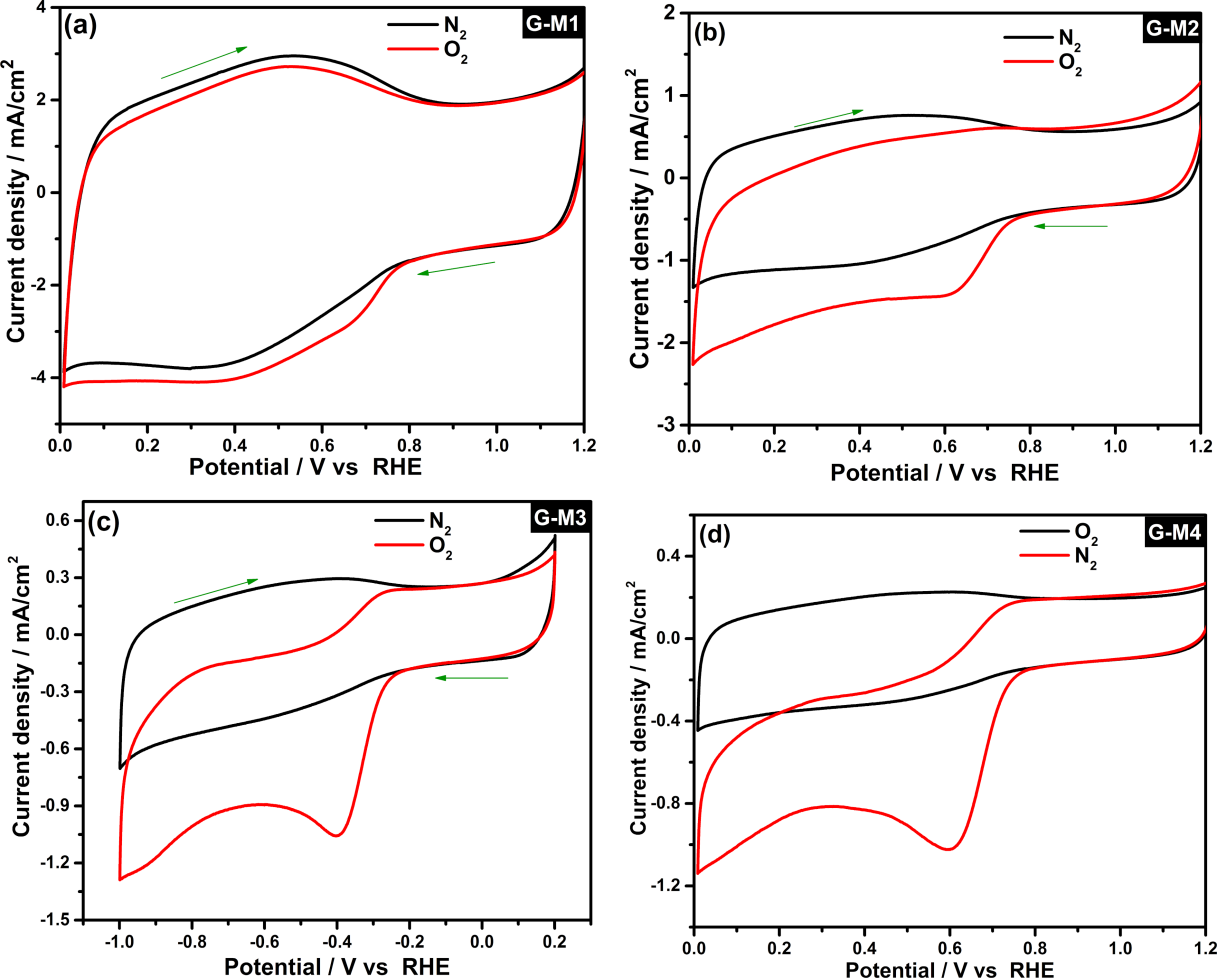
The CV profiles of different electrodes in 0.1 M KOH electrolyte saturated with N_2_ and O_2_. The current density is calculated using the geometrical surface area.

The ORR activity of different EEG samples is estimated using a conventional three-electrode system. The CV profiles of EEG-modified GCE in 0.1 M KOH electrolyte saturated with N_2_ and O_2_ gas are displayed in Figure 3. The electrodes exhibit capacitive (double layer) behavior in N_2_-saturated electrolyte, while a sharp reduction peak corresponding to oxygen reduction in O_2_-saturated electrolyte is shown in all the cases. The intensity of the peak (peak current density) corresponds to the ORR process and varies with the degree of oxygen functionalities (from G-M1 to G-M4), which reveals the effect of the degree of functionalization in ORR. The CV profiles plotted using the current densities calculated using the electrochemical surface area also shown similar trends, as shown in Supporting Information File 1, Figure S5 (the detailed procedure to calculate electrochemical surface area is given in the Supporting Information File 1, see Figure S6). To summarize, the G-M1 sample showed the highest reduction current under the same experimental conditions whereas G-M4 showed the lowest current with the rest of the samples in between these two samples.

**Figure 4:**
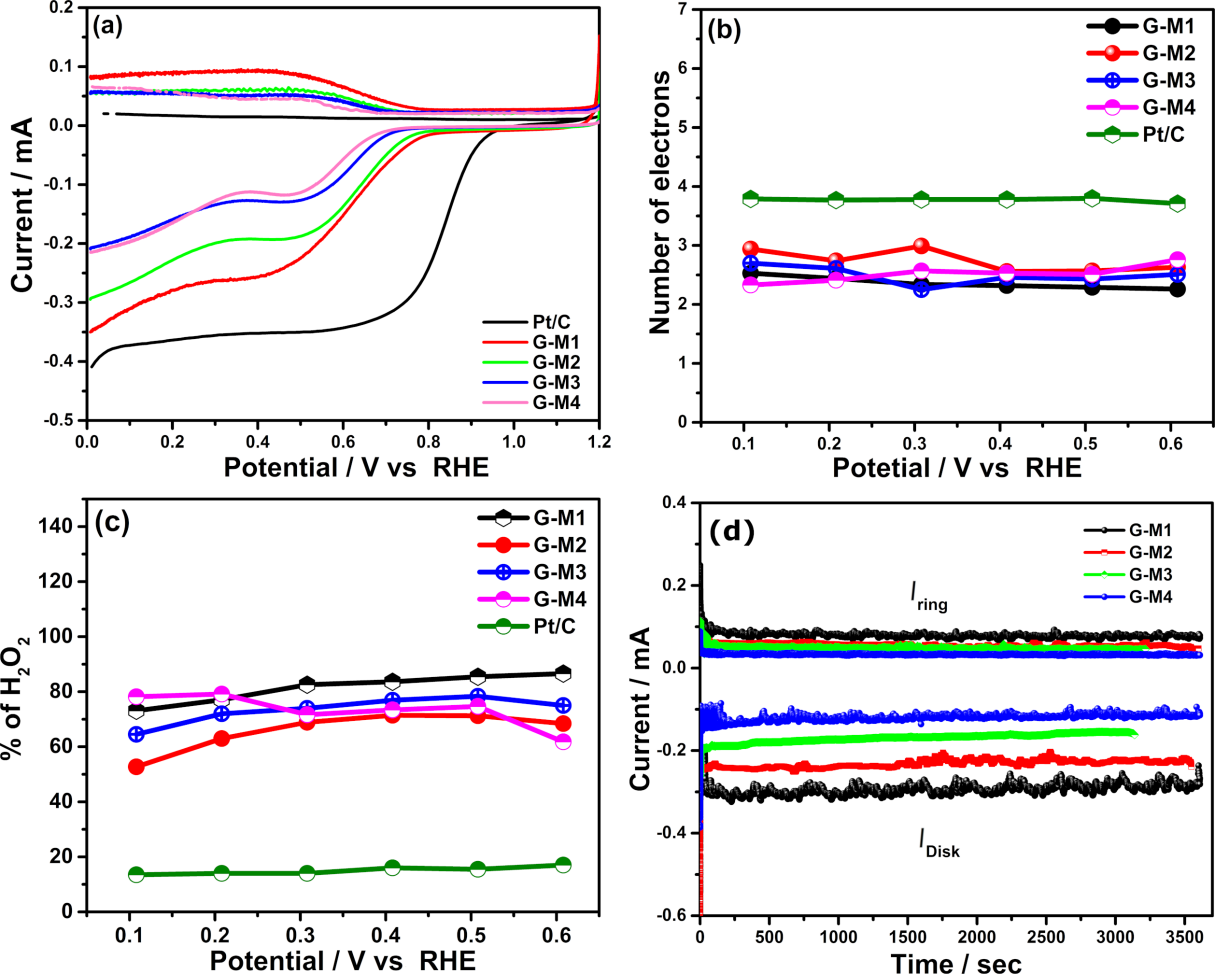
(a) LSV scans of O_2_ reduction and H_2_O_2_ (HO_2_^−^) oxidation on different EEG-modified RRDE samples. (b) The number of electrons transferred during ORR per O_2_ molecule and (c) the percentage (%) of formed H_2_O_2_ (calculated from Figure 4a) is shown. (d) Chronoamperograms of O_2_ reduction and H_2_O_2_ oxidation processes at 0.358 V and 1.5 V vs RHE on different electrodes.

To understand the ORR reaction pathway, rotating ring and disk electrode (RRDE)-based hydrodynamic voltammetry is conducted. Figure 4a shows ORR linear sweep voltammetry (LSV) scans of different EEG samples on GCE disk (4 mm diameter) and the H_2_O_2_ oxidation over the platinum ring (potential kept at 1.5 V vs RHE) at 10 mV/s scan rate at 1600 rotation per minute (rpm) for the electrode. The experiments are also carried out at different rotation speeds, and the data is given in Supporting Information File 1, Figure S7. The electrochemical parameters derived from these experiments (at 0.358 V vs RHE) are shown in Table 1. As shown in the Figure 4, the current density and onset potential of the reaction vary with the degree of (oxygen) functionalization. A benchmark Pt/C catalyst performance is also shown for comparison.

**Table 1:** The RRDE experiment electrochemical parameters obtained at 0.358 V (vs RHE, from Figure 4a).

Active materials	Disk current (mA)	Ring current (mA)	Onset potential (V)	Number of electrons (*n*)	% H_2_O_2_ at 0.358 V

G-M1	0.24	0.067	0.86	2.35	82.07
G-M2	0.18	0.046	0.84	2.41	79.09
G-M3	0.12	0.031	0.81	2.43	78.43
G-M4	0.11	0.028	0.77	2.40	80.01
Pt/C	0.32	0.015	1.01	3.65	17

The onset potential of ORR is found to become more favorable with a high degree of functionalization, reaching 860 mV for the G-M1 electrode and 770 mV for the G-M4 sample. The onset potential and current density of G-M2 and G-M3 are found to be between those of G-M1 and G-M4. Interestingly, the difference in fluorine content (which is nominal) did not affect the onset potential of the ring current. The shape of the LSV scans shows a two-step in reduction, which is due to the reduction of oxygen to H_2_O_2_ at lower overpotential and its further reduction to H_2_O at higher potentials [44]. This suggests that the ORR proceeds through a two-electron reduction path in EEG samples while the sharp Faradaic current enhancement followed by a plateau in Pt/C shows that it is a one-step reduction process.

The quantity of H_2_O_2_ produced is analyzed using a platinum ring electrode at 1.5 V vs RHE. The ring current is higher (0.067 mA) for highly functionalized graphene and less (0.028 mA) for less functionalized (G-M4) graphene, which displays higher peroxide formation in G-M1. Hence G-M1 shows higher O_2_ reduction current and higher peroxide oxidation than the others because the oxygen functionalities possess selective activity towards ORR, which is in line with the other recent reports [37]. The number of electrons transferred per oxygen molecule and percentage (%) of H_2_O_2_ produced during ORR are calculated (using the details given in Supporting Information File 1), and the data are shown in Figure 4b and 4c, respectively.

Table 1 gives the kinetic parameters calculated from Figure 4a (LSV of ORR on RRDE) at 0.358 V vs RHE. As shown in the table, all the EEG samples, irrespective the degree of functionalization, show the number of electrons transferred as ≈2.4 electrons per oxygen and yield ≈80 ± 2% H_2_O_2_ generation. Figure 4c shows the number of electrons transferred over a potential range from 0.608 to 0.108 V vs RHE. Interestingly, the ORR follows the peroxide reduction path (about 2.2 ± 0.1 electron per oxygen molecule at 0.4V vs RHE) at lower overvoltage, and the slight increase can be observed in the number of electrons transferred at higher overpotential. The increase in the number of electrons can be attributed to fluorine functional groups attached to graphene, which can undergo a direct 4-electron path at higher overpotential, as reported previously [47,55]. Highly fluorinated EEG (G-M2) shows about three electrons per oxygen at higher overpotential (0.3008 to 0.108 V). However, it is found that the fluorine content can be reduced by reducing the concentration of the electrolyte, which further improves the selective production of H_2_O_2_ even at relatively higher overpotential.

In order to test the stability of these catalysts, chronoamperometry experiments were conducted using RRD electrodes where the disk current was kept at 0.358V vs RHE and the ring potential was kept at 1.5 V vs RHE. Figure 4d depicts the chronoamperograms of the ORR at the disk and H_2_O_2_ oxidation at the ring electrode for 1 h at 1600 rpm, which shows reasonable stability in the current over time (both electrodes). Furthermore, the variation in the current at the ring and disk for different EEG samples follows the same trend as that found in the LSV scans (Figure 4a). The stability of the EEG-based catalysts is also tested by repeated cycling of LSV at 1600 rpm for 1000 cycles (at 100 mV·s^−1^). The LSV scans of ORR before and after 1000 cycles are given in Supporting Information File 1, Figure S8 and the results show that all the EEG samples, irrespective of the degree of functionalization, display very little degradation in performance. This indicates that the functionalized EEG samples are stable electrocatalysts for electrochemical H_2_O_2_ production even under harsh alkaline conditions.

**Figure 5:**
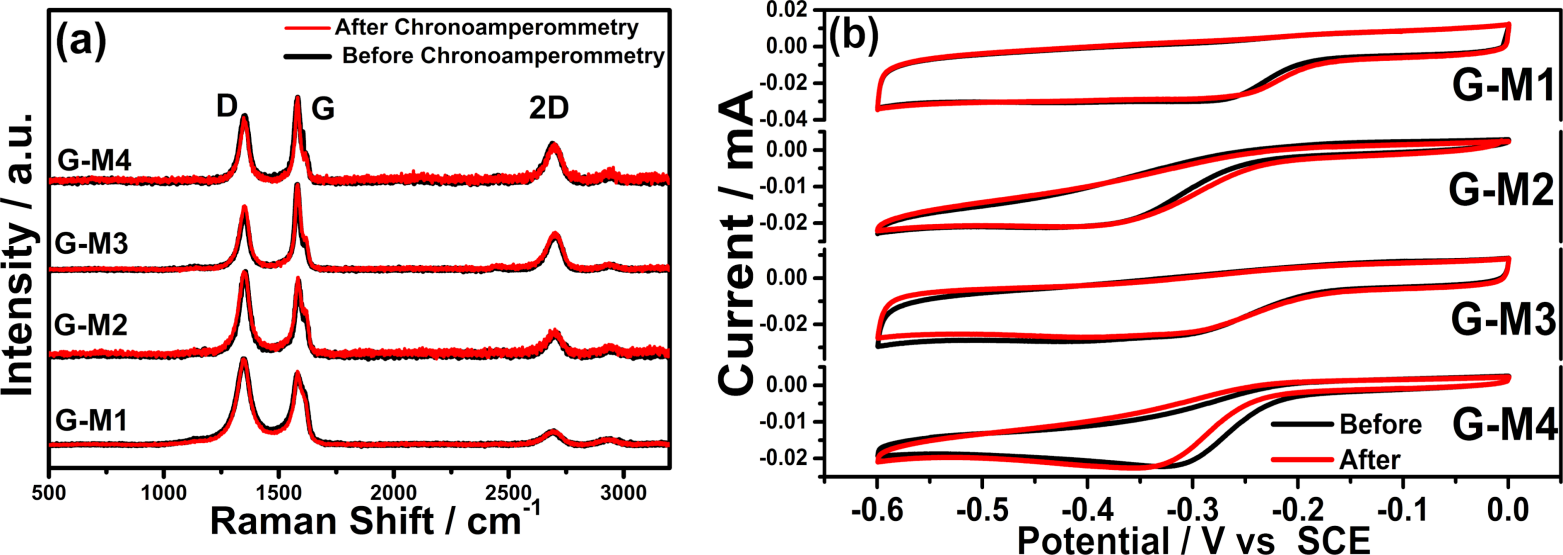
(a) Raman spectra of EEG samples before and after 3 h of chronoamperometry in 0.5 M H_2_SO_4_ solution at 0.45 V vs RHE. (b) ORR in 0.1 M KOH solution before and after chronoamperometry.

Hence it can be concluded that the presence of oxygen functional groups is a key factor in improving the ORR, and they undergo the redox process during the reaction in acidic medium (see Figure 2a). In order to test the durability of the materials in acidic medium, chronoamperometry studies were carried out in 0.5 M H_2_SO_4_ at 0.45 V vs RHE for 3 h. The EEG samples were studied (before and after chronoamperometry) using Raman and FTIR spectroscopy along with the electrochemical performance in 0.1 M KOH solution, and the data is given in Figure 5. In Figure 5a, the Raman spectra of EEG before and after 3 h of chronoamperometry are shown, which show no appreciable change in either the peak position or peak intensity. This indicates that no additional defects are created during the experiment. The important Raman peaks are marked in the figure and the shoulder peak in “G” is due to the additional single phonon intra-valley scattering process (named as D’) which is due to the presence of defects. However, the FTIR spectrum (see Figure S4b) shows evidence for the formation of OH functional groups during the chronoamperometry, which is suggests that most of the C=O functional groups are converted into –C–OH during the reaction. To study the effect of the change in the functionalization on ORR, we recorded the CV of ORR in 0.1 M KOH solution before and after chronoamperometry. Such changes are found to have an insignificant effect on the performance of the material towards ORR (see Figure 5b).

**Figure 6:**
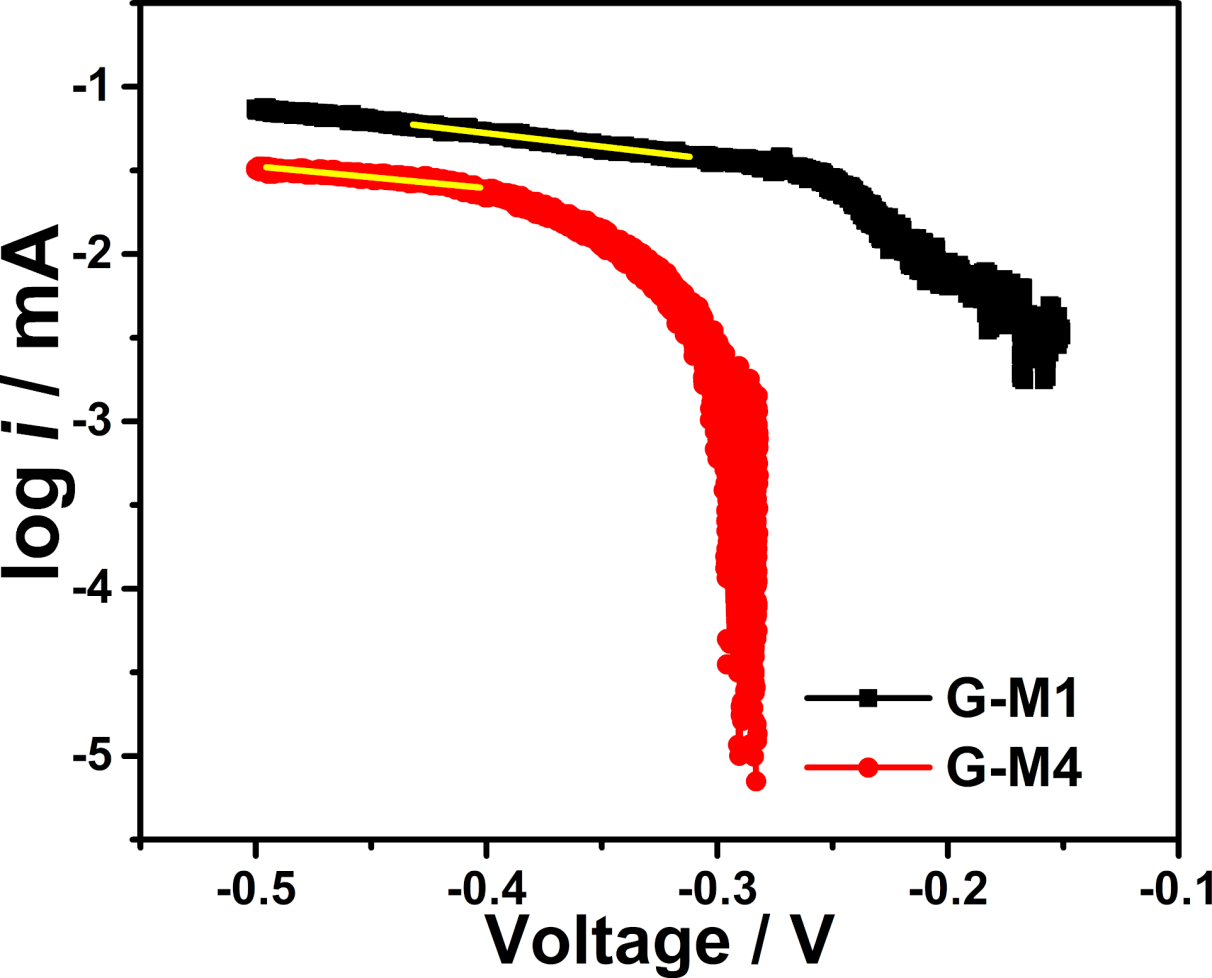
Tafel plots of ORR over EEG-modified electrodes in 0.1 M KOH O_2_-saturated electrolyte.

To evaluate the EEG samples further, the kinetic parameters such as rate constant and exchange current density are calculated from the Tafel plot analysis (in O_2_-saturated electrolyte) and are shown in Figure 6. As expected, G-M1 shows a high exchange current 1.2 × 10^−5^ A, and G-M4 shows a low exchange current 7.4 × 10^−6^ A, which demonstrates that G-M1 is kinetically more favorable towards ORR than G-M4. The rate constant (*k*) was calculated from the following equation [56], which is derived from the Butler–Volmer model [56]:

[2] $$i_{\text{o}}=FAk^{0}C_{\text{O}}^{*}{}^{\left(1-\alpha \right)}C_{\text{R}}^{*}{}^{\alpha }$$

where *i*_o_ is the exchange current density, *F* is the Faraday constant (96485 C), 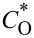 is the concentration of the oxidant, 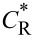 is the concentration of the reductant, α is the transfer coefficient, *A* is the surface area of the electrode (0.07 cm^2^), and *k*^0^ is the heterogeneous rate constant. In this case, we considered *C*_O_ = *C*_R_ = *C*, where *C* is the concentration of the dissolved oxygen which is 1.26 × 10^–6^ mol·cm^−3^ [57–58]. The rate constant value calculated from the above equation is 1.39 × 10^−3^ cm·s^−1^ for G-M1 and 8.52 × 10^−4^ cm·s^−1^ for G-M4 which follow the trend observed in exchange current density. Both *i*_o_ and *k*^0^ emphasize the importance of the quantity of functional groups in ORR.

**Figure 7:**
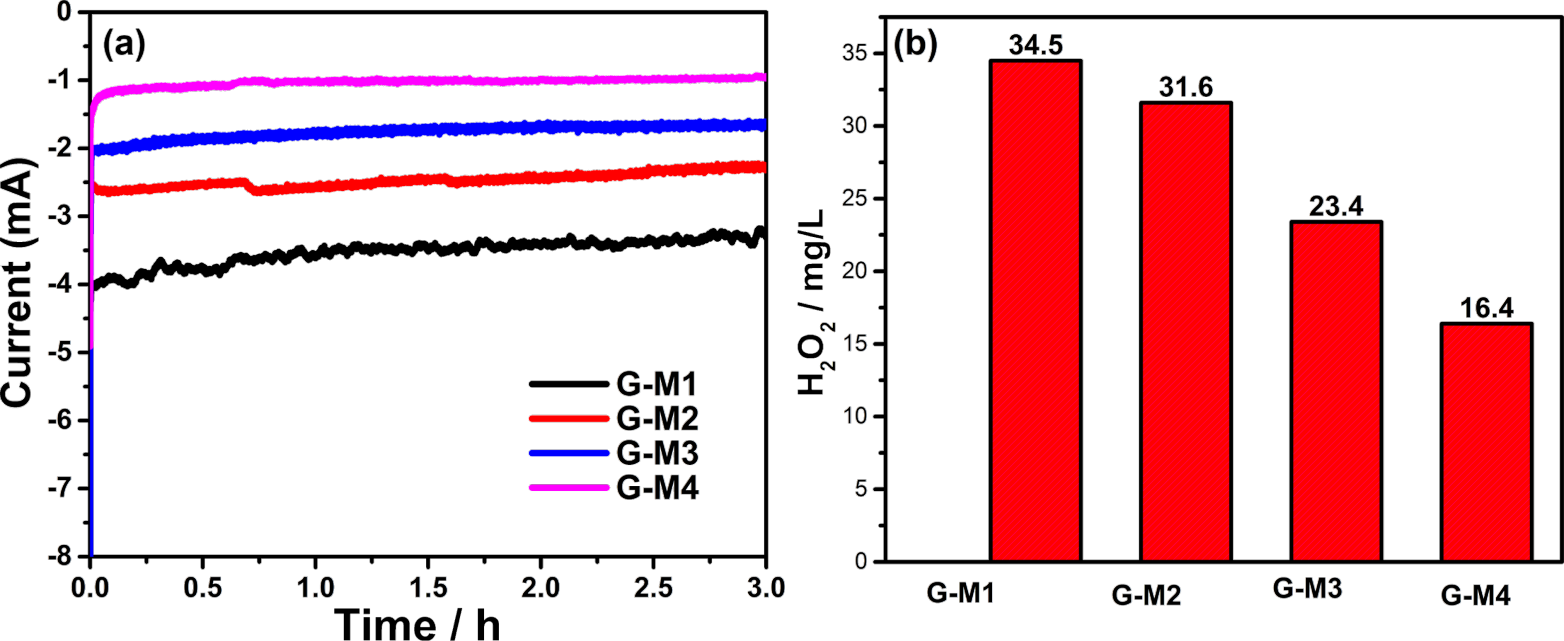
(a) Chronoamperogram of O_2_ reduction at 0.358 V on graphite paper modified with EEG. (b) The amount of H_2_O_2_ produced by each sample is shown in Figure 7b.

Large-scale production of peroxide using these EEG samples are conducted using bulk electrolysis. EEG-coated and uncoated graphite paper was employed for the electrochemical production of H_2_O_2_. The H_2_O_2_ produced through ORR is also quantified using a Ce(SO_4_)_2_ solution assisted UV–visible absorbance based analysis, where the details and calibration are given in Supporting Information File 1. Figure 7a shows chronoamperograms of ORR on EEG-coated graphite papers at 0.358 V vs RHE in 0.1 M KOH electrolyte for 3 h. G-M1 shows the highest current where the current subsequently reduced from sample G-M1 to G-M4. The amount of H_2_O_2_ produced is calculated as 34.5, 31.6, 23.4, and 16.4 mg/L for G-M1, G-M2, G-M3, and G-M4, respectively. The high quantity of H_2_O_2_ production in G-M1 can be attributed to its high degree of oxygen functional groups, particularly due to C=O. The amount of peroxide formed by this method is found to be higher or on par with recent reports [3]. This opens an efficient pathway for the single-step large-scale production of peroxide-generating carbon-based ORR catalysts.

The weight of the anode (graphite electrode) used for the electrochemical exfoliation was ≈0.374 g and the 1–2 h of exfoliation resulted in the complete consumption of graphite, delivering ≈0.180 ± 0.005 g of exfoliated functionalized graphene. Hence the average yield of this process was found to be ≈45%.

Hence such chemically modified graphene powders, which are proven to be dispersible in a variety of organic solvents [59], offer alternate possibilities towards existing metal-based peroxide generation technologies. Controlling the electronic properties via the thermal reduction method can further tune the charge transfer properties of such functionalized graphene powders [60], opening a plethora of opportunities in this field.

## Conclusion

An efficient single-step method (without any post-treatment) has been developed for the high-yield synthesis of carbon-based peroxide, generating ORR catalysts having varying functionalities. The role of quinone-containing functional groups in graphene towards the electrochemical ORR process is unraveled, and the higher C=O content results in a large amount of H_2_O_2_ production at a high rate. The other functionalities in graphene such as fluorine have a minimal role in lower potential reduction reactions while they become prominent at higher potentials, where they undergo a direct four-electron reduction of O_2_ to water. The concentration of the electrolyte was found to be related to the thickness of the formed exfoliated graphene and its functionalities, and the 1 M KF-based exfoliation resulted in ultrathin layers for the sample G-M1, which had a high amount of C=O groups but fewer C–F functionalities. These electrochemically exfoliated functionalized graphene samples are found to be highly stable in alkaline electrochemical conditions, whereby 3 h of ORR produced ≈34 mg/L of peroxide for an applied potential of 0.358 V vs RHE, indicating a production on par or higher than the recently reported state-of-the-art catalysts [3].

## Experimental

### Materials

Graphite rods of 3 mm diameter and 150 mm length with 99.999% purity, sodium hydroxide (ACS grade), sulfuric acid, and hydrogen peroxide (27% w/v) were obtained from Alfa Aesar. Nafion solution (5% w/w) was purchased from Sigma-Aldrich, and potassium fluoride (KF) and Ce(SO_4_)_2_ were obtained from Sisco research laboratories, India. All of the high purity chemicals were employed as-received without any further purification. Ultrahigh purity oxygen (99.999%) was used for electrochemical reactions.

### Synthesis of electrochemically exfoliated graphene (EEG)

EEG was synthesized via a method reported previously [42–43], where the degree of functionalization can be tuned by changing the concentration of the electrolyte (KF) from 1 to 4 M. In this process, two graphite rods were used as electrodes in KF aqueous electrolyte having different concentrations. A regulated DC power supply (Physitech electronics, model: PHY8230) in galvanostatic mode (0.2 A/cm^2^) was employed to carry out the electrochemical exfoliation. After the complete consumption of the graphite rod (anode), a black precipitate was collected from the electrolyte through centrifugation and subsequently washed with 1 M H_2_SO_4_ followed by deionized water until the solution becomes neutral pH. The powder was dried at 60 °C for 12 h and used for further studies. The samples prepared in 1 M, 2 M, 3 M, and 4 M KF electrolytes were named as G-M1, G-M2, G-M3, and G-M4, respectively.

### Characterization

As prepared EEG samples were characterized using a Renishaw Invia Raman spectrometer with a 532 nm laser as the excitation source. XPS (Thermo Scientific EASCA lab 2000) and FTIR spectroscopy were used to unravel the nature and degree of functionalization along with the change in morphology of these samples. The surface area of the samples was analyzed using Brunauer–Emmett–Teller (BET) adsorption isotherms from a Quantachrome Nova 1200e surface area analyzer. AFM was used to study the thickness of the exfoliated layers.

### Electrochemical experiments

All of the electrochemical ORR experiments were carried out in a conventional three-electrode system with a catalyst ink modified GCE as a working electrode, Hg/Hg_2_Cl_2_ and platinum foil (results were cross-checked with graphite rod counter electrode, too) used as a reference and counter electrodes, respectively. The electrochemical performance of the materials was analyzed using cyclic voltammetry (CV), linear sweep voltammetry (LSV), and rotating ring and disk electrode (RRDE) measurements. A BioLogic SP-300 instrument was used for these controlled experiments and an RRDE with a GC disk (4 mm diameter) and Pt ring (5 mm and 7 mm outer and inner diameters, respectively) electrode was employed for the RRDE-based experiments. The catalyst ink for the electrochemical characterization was prepared by dispersing 10 mg of functionalized graphene in 375 µL of the solvent mixture consisting of isopropyl alcohol (IPA, 275 µL), water (50 µL), and *N*,*N*-dimethyl formamide (DMF, 50 µL). 3 µL and 5 µL of the above-prepared ink was drop cast over the well-polished GCE and RRDE (GCE disk having 4 mm diameter), respectively. All of the electrochemical experiments were carried out in 0.1 M KOH solution (for ORR process) and 0.5 M H_2_SO_4_ solution (for electrochemical surface area measurements). The 0.1 M KOH electrolyte was initially saturated with ultrahigh pure N_2_ followed by O_2_ gas before the respective analysis. The details of the calculation for the number of electrons transferred are given in Supporting Information File 1.

The quantification of the H_2_O_2_ produced was carried out using a UV–vis spectrometer [37,61]. A calibration curve was made using 1 mM Ce(SO_4_)_2_ (25 mL) solution, 1% H_2_O_2_ solution (30% H_2_O_2_ solution was diluted to 1%) and the details can be found in Supporting Information File 1 (Figure S9) [61]. Bulk electrolysis was conducted using the above-mentioned inks. In brief, 100 µL of the above-prepared catalyst ink was drop cast over a 1 cm^2^ area of the Toray carbon paper having 100 mm length and dried at room temperature. These electrodes were used as working electrodes for bulk electrolysis wherein the electrochemical cell constitutes 100 mL of O_2_-saturated 0.1 M KOH electrolyte and a constant potential of 0.358 V vs RHE, applied for 3 h. Subsequently, the sample (3 mL of electrolyte) was collected and used for the quantification of the H_2_O_2_. The details of the quantification are given in Supporting Information File 1.

## Supporting Information

The supporting information includes additional TEM images, a detailed description of the quantification of H_2_O_2_, deconvoluted XPS spectra of O 1s, AFM analysis, FTIR spectra, ECSA calculations, and RRDE analysis for all EEG samples.

File 1 Additional experimental results and analysis.
