# Supplementary material for: Electrochemically derived functionalized graphene for bulk production of hydrogen peroxide
Source: Beilstein J Nanotechnol. 2020 Mar 9;11:432–42. doi: 10.3762/bjnano.11.34 (PMC7082696; doi:10.3762/bjnano.11.34)
Supplement: File 1 — Additional experimental results and analysis. [file Beilstein_J_Nanotechnol-11-432-s001.pdf]

BEILSTEIN JOURNAL OF NANOTECHNOLOGY

Supporting Information

for

Electrochemically derived functionalized graphene for bulk production of hydrogen peroxide

Munaiah Yeddala, Pallavi Thakur, Anugraha A and Tharangattu N. Narayanan

Beilstein J. Nanotechnol. **2020,** *11,* 432–442. doi:10.3762/bjnano.11.34

Additional experimental results and analysis

License and Terms: This is a supporting information file under the terms of the Creative Commons Attribution License (https://creativecommons.org/licenses/by/4.0). Please note that the reuse, redistribution and reproduction in particular requires that the authors and source are credited.
The license is subject to the *Beilstein Journal of Nanotechnology* terms and conditions: (https://www.beilstein-journals.org/bjnano)

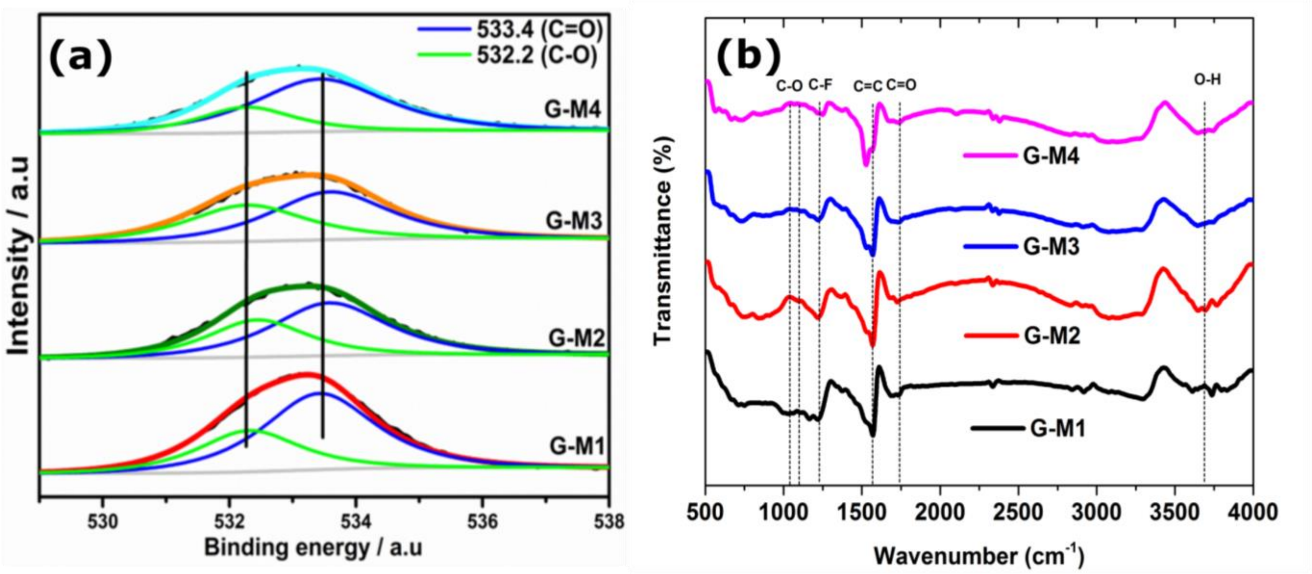

Figure S1: (a) Deconvoluted high resolution XP spectra of O 1s. (b) FTIR spectra of EEGs.

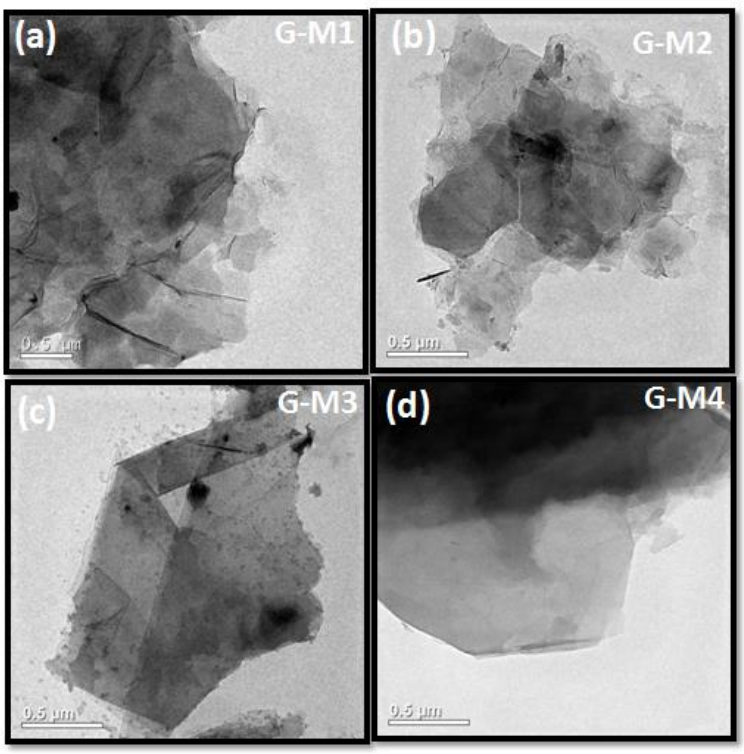

Figure S2: TEM images of G-M1, G-M2, G-M3, and G-M4 showing the electron transmission variation from M1 to M4.

Table S1: Elemental composition of carbonyl and alcoholic oxygen in total oxygen functional groups calculated from Figure S2(a).

	% of C=O	% of C-O
G-M1	67.8	32.2
G-M2	62.0	38.0
G-M3	56.2	43.8
G-M4	72.8	27.2

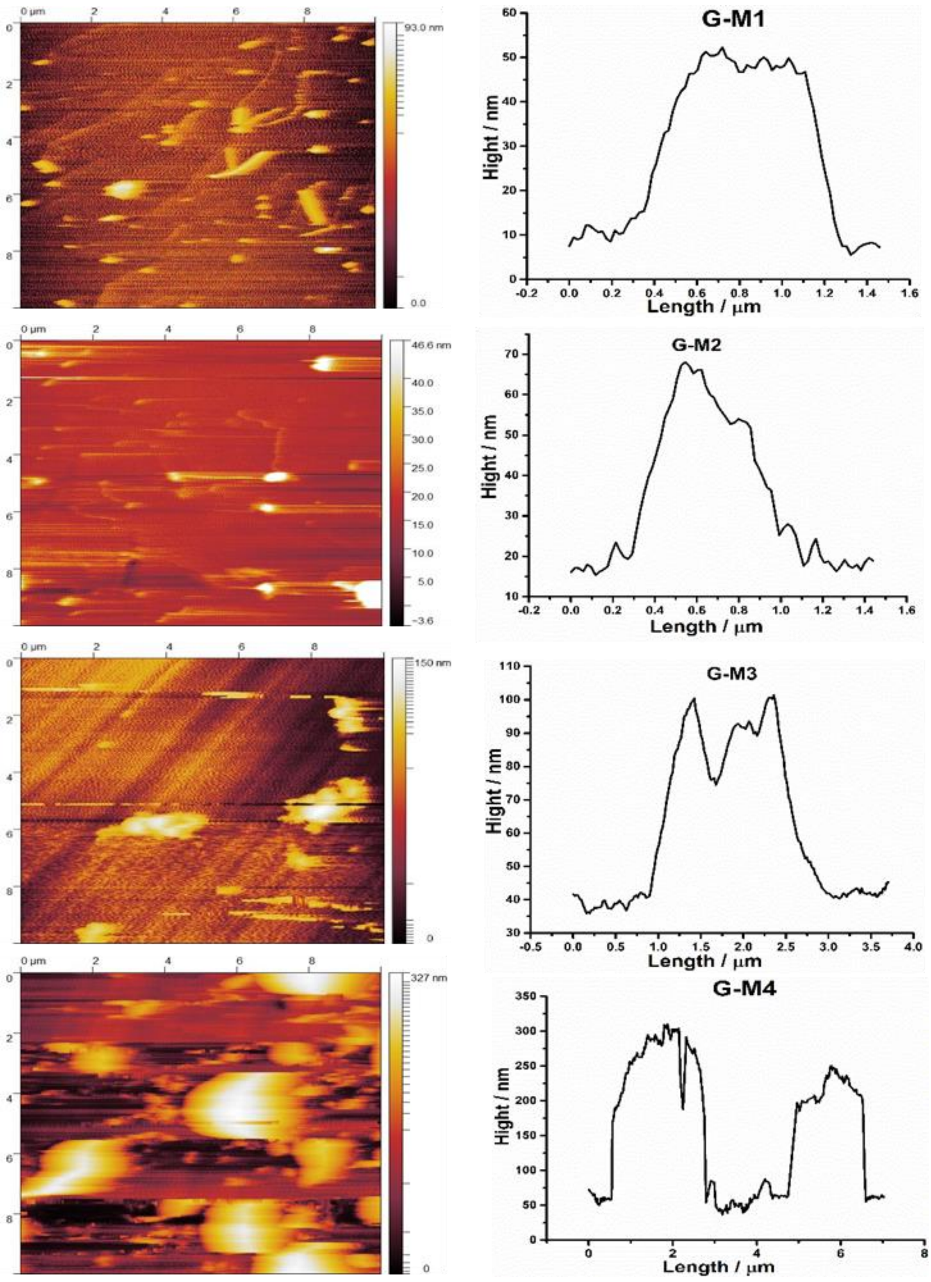

Figure S3: AFM images of EEGs and their corresponding line profiles showing the thickness variations are shown.

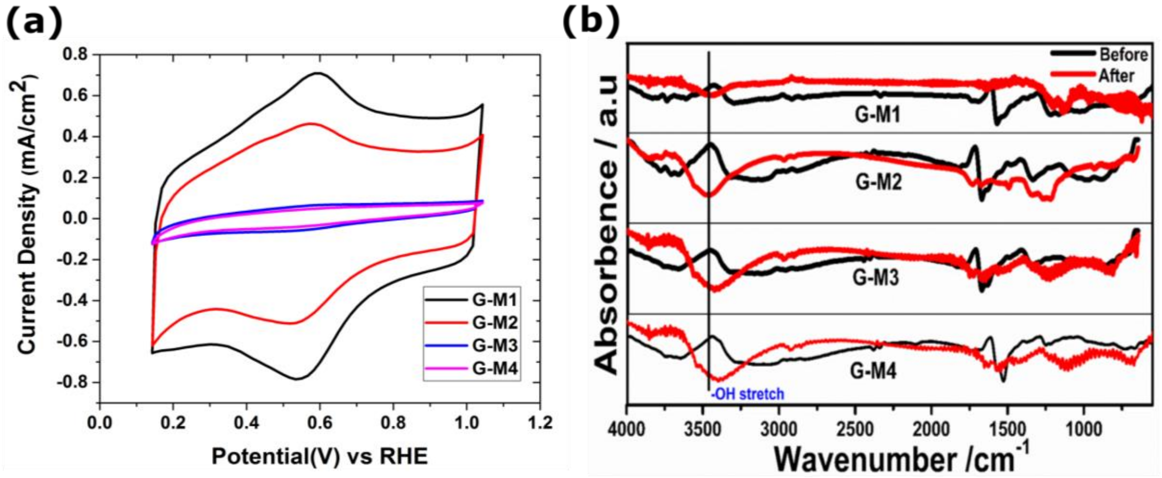

Figure S4: (a): The CVs of different EEGs in (a) acidic (0.5 M H_2SO_4) medium at 5 mV/s scan rate. (b) FTIR spectra of EEGs before (black) and after (red) 3 h of chronoamperometry in 0.5 M H_2SO_4 solution. The intensity of all the spectra are normalized for comparison.

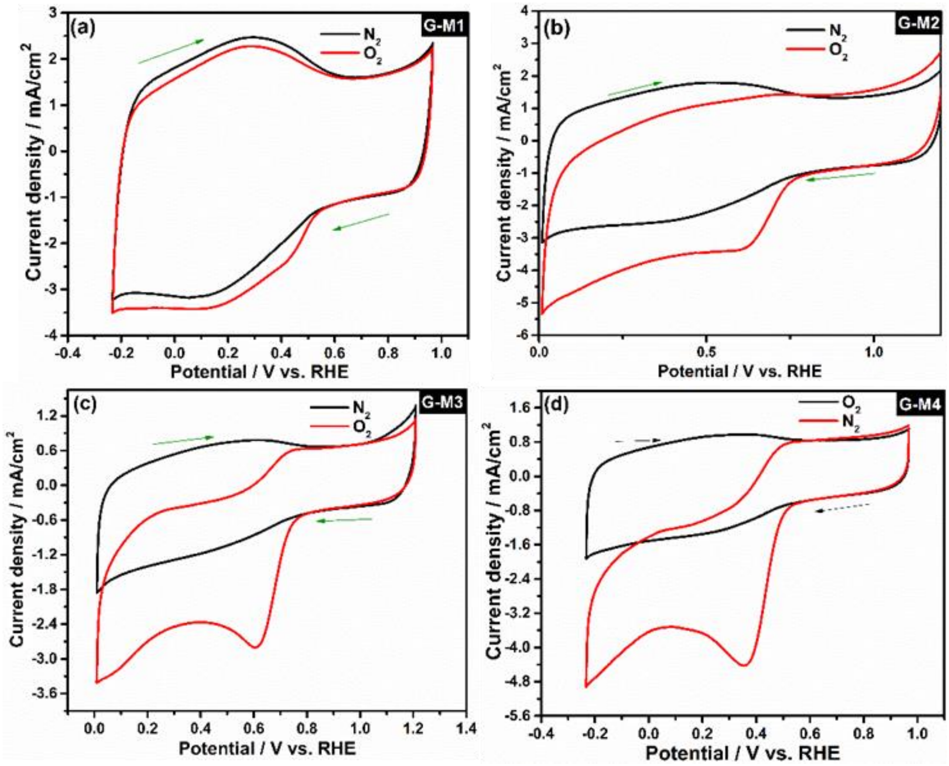

Figure S5: CVs of ORR in N_2 and O_2 saturated 0.1 M KOH electrolyte at 10 mV/s scan rate. The current density values are calculated with respect to ECSA of the materials.

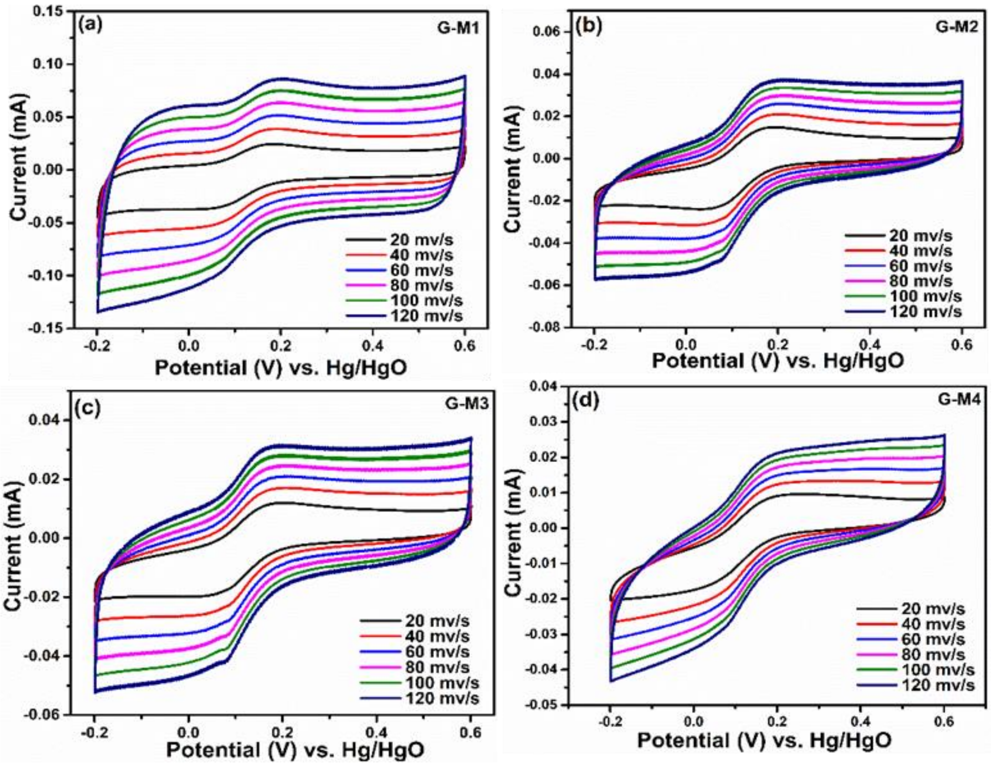

Figure S6: Ferri/ Ferro redox responses of EEG modified GCEs at different scan rates.

Randles-Sevcik equation is used to calculate the electrochemical surface area:

$I_{p=}$ $(2.69*10^5)\ n^{3/2}A\ D^{1/2}v^{1/2}C$

I_p=m $v^{1/2}$

Where i_p= peak current (A)

D = Diffusion coefficient in cm^2/s of potassium ferricyanide solution

$v^{1/2}$ = Sqrt. Of scan rate (V/s)

C = concentration of electrolyte (5 mM)

A = ECSA

m = Slope

ECSA

M1 = 0.0837 cm^2 for 0.05 mg loading

M2 = 0.0297 cm^2 for 0.05 mg loading

M3 = 0.0264 cm^2 for 0.05 mg loading

M4 = 0.0162 cm^2 for 0.05 mg loading

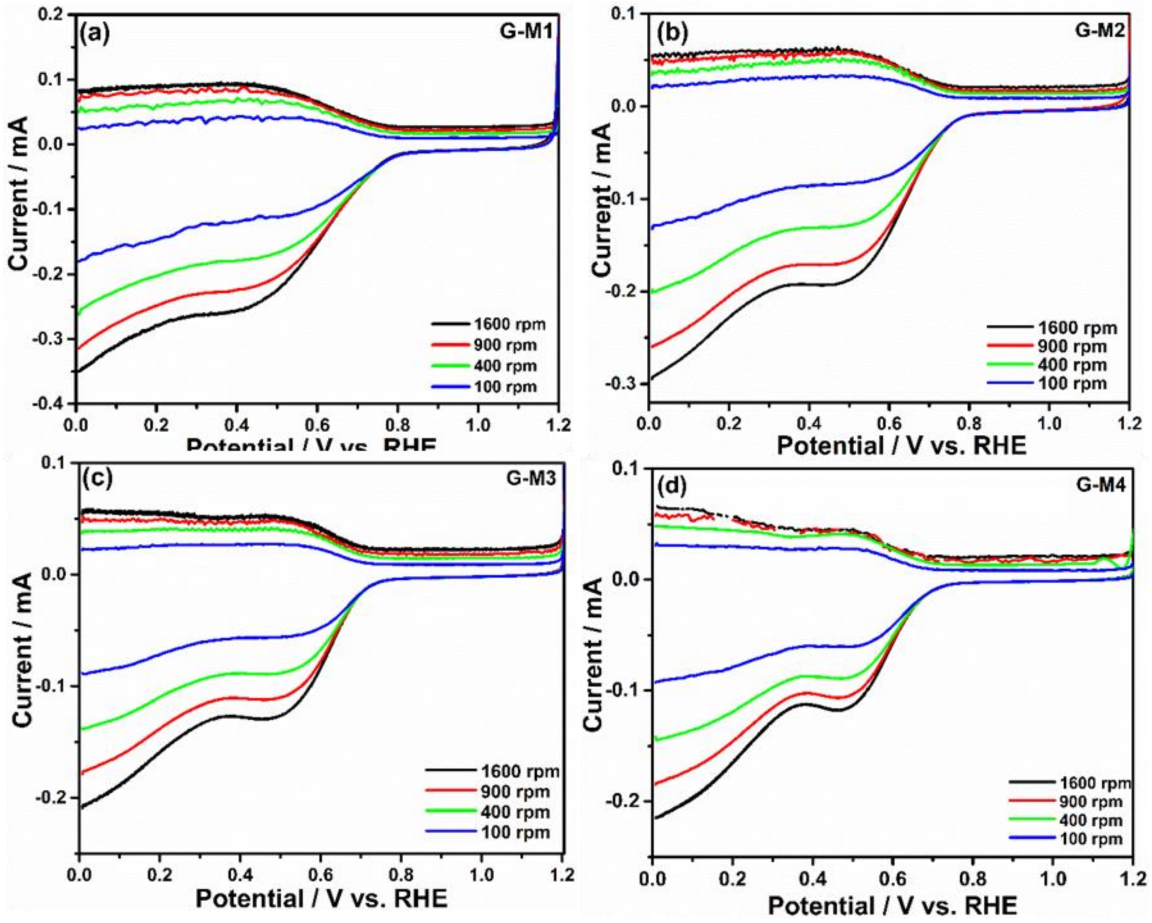

Figure S7: LSVs of ORR on Rotating disk modified with EEGs at 10 mV/s and oxidation of H_2O_2 at Pt ring electrode at 1.5 V *vs*. RHE at different rotation rates.

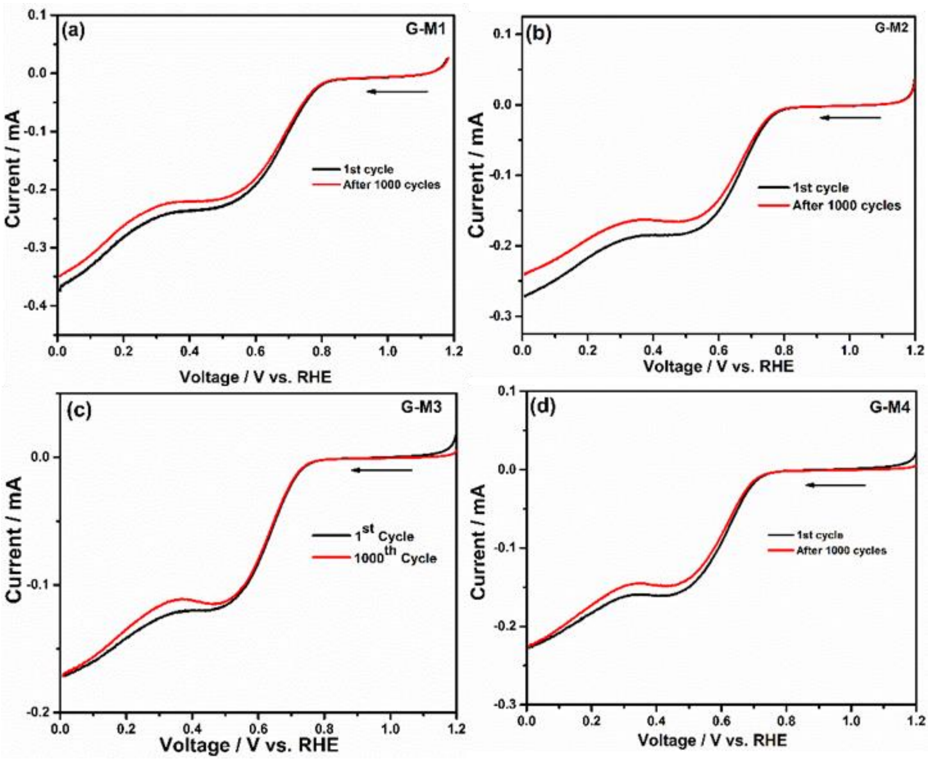

Figure S8: LSVs of ORR in 0.1 M KOH electrolyte at 100 mV/s scan rate and 1600 rpm rotation before and after 1000 repeated LSV. The samples show no considerable change either in onset potential or in current. No shift can be seen in half wave potential too.

Calculation of number electrons transferred and % of H_2O_2 produced

Some of the critical electrochemical parameters such as the number of electrons transferred per oxygen molecule during the reduction can be calculated from either from Koutecky-levich (K-L-Plots) equation or from RRDE electrochemical data. Since RRDE electrochemical data is more reliable than that of K-L plots, we employed RRDE data to calculate the number of electron and % of H_2O_2 produced during the ORR [3].

Ring and disk current in RRDE method employed as follows (equation 1) to determine the number of electrons transferred during ORR

$$n = \frac{4I_D}{I_D + \frac{I_R}{N}}$$ ----------------(1)

Where I_D and I_R are disk and ring currents at a given potential respectively and N is the collection efficiency of the ring which is 0.4 determined using Fe^{2+}/Fe^{3+} standard redox couple.

The % of H_2O_2 formed at GC disk and oxidized at the ring is calculated using the following equation.

$$H_2O_2 (\%) = 200 \times \frac{\frac{I_R}{N}}{\frac{I_R}{N} + I_D}$$ ------------ (2)

Where I_R and I_D are ring and disk currents and N is the collection efficiency of the ring (0.39).

<u>Quantification of H_2O_2</u>

1 mM $Ce(SO_4)_2$ solution was prepared by dissolving 33.2 mg of salt in 100 mL of 0.5 M H_2SO_4 solution [1]. UV spectrum of 1 mM $Ce(SO_4)_2$ solution was recorded which shows a sharp peak at 325 nm (Figure S9a). 3 µL of 1% H_2O_2 (30% H_2O_2 was diluted to 1% by the addition of water) was added to 25 mL of the above solution to convert Ce^{4+} into Ce^{3+} which show a different peak in UV spectra [2]. Consecutive addition of H_2O_2 increase the concentration of Ce^{3+} in the solution which can be seen in UV spectra. UV spectrum of Ce^{3+} shows a series of Peaks between 200–275 nm and the peak at 254 nm is independent of the concentration of the SO_4^{2-} ions in the solvent [2]. The area under the curve is directly proportional to the concentration of Ce^{3+} ion. The peak at 254 nm is considered for the construction of the calibration curve and the calibration curve is shown in the figure S9b.

The quantification of H_2O_2 generated during chronoamperometry was determined from UV spectra of $Ce(SO_4)_2$ (Figure S9c) after addition of 3 mL of 0.1 M KOH (after 3 h of chronoamperometry at 0.358 V to 25 ml of 1 mM $Ce(SO_4)_2$ solution. The area

under the peak at 254 nm is calculated to determine the concentration of H_2O_2 from calibration curve.

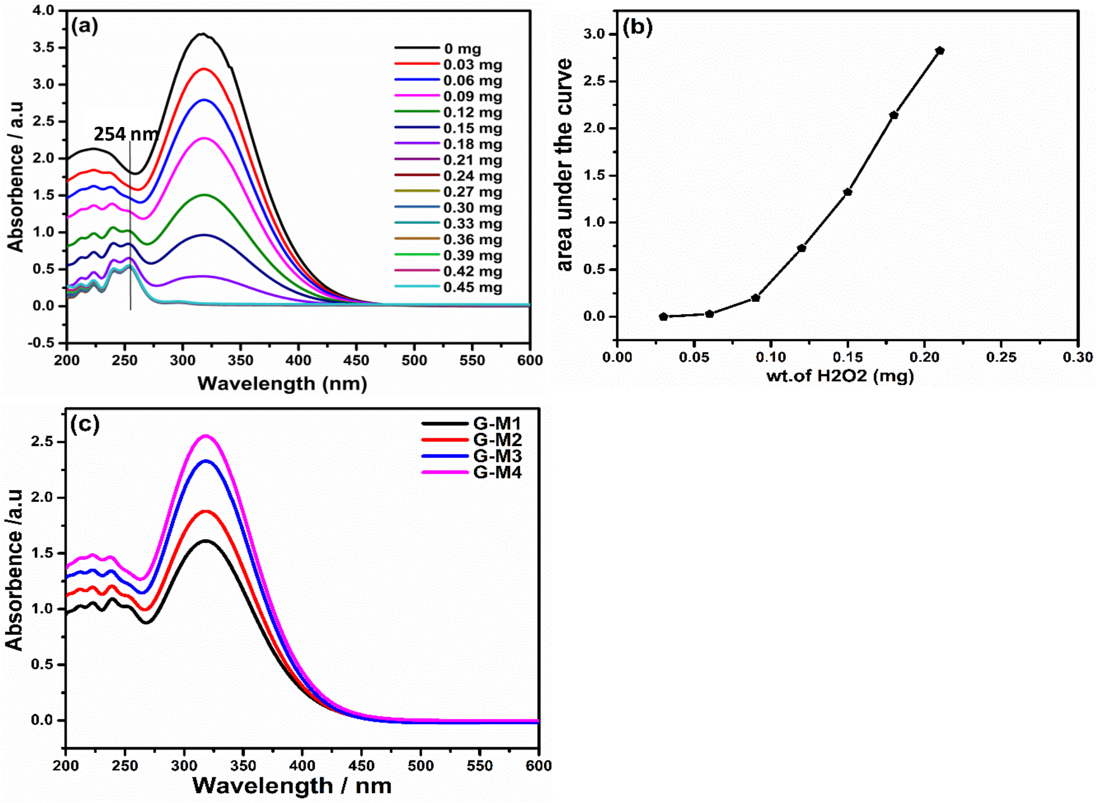

Figure S9: (a) Ultraviolet absorption spectrum $Ce(SO_4)_2$ solution having different concentrations of H_2O_2. (b) Calibration curve derived from Figure S9a. (c) Ultraviolet absorption spectrum $Ce(SO_4)_2$ solution (25 mL) having 0.1 KOH solution (3ml) after 3 h of chronoamperometry at −0.358 V having EEGs modified carbon paper as working electrode.

References

1. Lu, Z.; Chen, G.; Siahrostami, S.; Chen, Z.; Liu, K.; Xie, J.; Liao, L.; Wu, T.; Lin, D.; Liu, Y.; Jaramillo, T. F.; Nørskov, J. K.; Cui, Y. High-efficiency oxygen reduction to hydrogen peroxide catalysed by oxidized carbon materials. *Nature Catalysis* **2018**, *1*, 156-162.

2. Greenhaus, H. L.; Feibush, A. M.; Gordon, L. Ultraviolet Spectrophotometric Determination of Cerium(III). *Analytical Chemistry* **1957**, *29*, 1531-1534.

3. Zhou, R.; Zheng, Y.; Jaroniec, M.; Qiao, S.-Z. Determination of the Electron Transfer Number for the Oxygen Reduction Reaction: From Theory to Experiment. *ACS Catalysis* **2016**, *6*, 4720-4728.
